# Genetic risk score to predict biochemical recurrence after radical prostatectomy in prostate cancer: prospective cohort study

**DOI:** 10.18632/oncotarget.18275

**Published:** 2017-05-26

**Authors:** Jong Jin Oh, Seunghyun Park, Sang Eun Lee, Sung Kyu Hong, Sangchul Lee, Tae Jin Kim, In Jae Lee, Jin-Nyoung Ho, Sungroh Yoon, Seok-Soo Byun

**Affiliations:** ^1^ Department of Urology, Seoul National University College of Medicine, Seoul National University Bundang Hospital, Seongnam, Korea; ^2^ Department of Electrical and Computer Engineering, Seoul National University, Seoul, Korea; ^3^ School of Electrical Engineering, Korea University, Seoul, Korea; ^4^ Biomedical Research Institute, Seoul National University Bundang Hospital, Seongnam, Korea

**Keywords:** prostate cancer, genetic risk score, recurrence, predictive value

## Abstract

**Purpose:**

To investigate the genetic risk score (GRS) from a large-scale exome-wide association study as a tool of prediction for biochemical recurrence (BCR) after radical prostatectomy (RP) in prostate cancer (PCa).

**Results:**

The 16 SNPs were selected as significant predictors of BCR. The GRS in men experiencing BCR was -1.21, significantly higher than in non-BCR patients (–2.43) (*p <* 0.001). The 10-year BCR-free survival rate was 46.3% vs. 81.8% in the high-versus low GRS group, respectively (*p <* 0.001). The GRS was a significant factor after adjusting for other variables in Cox proportional hazard models (HR:1.630, *p <* 0.001). The predictive ability of the multivariate model without GRS was 84.4%, increased significantly to 88.0% when GRS was included (*p* = 0.0026).

**Materials and Methods:**

Total 912 PCa patients were enrolled who had received RP and genotype analysis using Exome chip (HumanExome BeadChip). Genetic results were obtained by the methods of logistic regression analysis which measured the odds ratio (OR) to BCR. The GRS was calculated by the sum of each weighted-risk allele count multiplied by the natural logarithm of the respective ORs. Survival analyses were performed using the GRS. We compared the accuracy of separate multivariate models incorporating clinicopathological factors that either included or excluded the GRS.

**Conclusions:**

GRS had additional predictive gain of BCR after RP in PCa. The addition of personally calculated GRS significantly increased the BCR prediction rate. After validation of these results, GRS of BCR could be potential biomarker to predict clinical outcomes.

## INTRODUCTION

Prostate cancer (PCa) is the most common malignant cancer affecting adult males in the United States, it was measured for 15 % of new cancer and 7 % of total male cancer related deaths in 2012 [1]. The radical prostatectomy (RP) is the gold standard surgical procedure for localized PCa [2]. Although localized PCa had good 10 year biochemical recurrence (BCR) outcomes which reported 73–99%, some PCa after RP will recurred [3, 4]. Five years after RP, 15% of men experience this biochemical recurrence (BCR), while 20% to 40% of men exhibit BCR 10 years after RP [5, 6]. Previous reports showed important predictors of BCR were serum prostate specific antigen (PSA), Gleason score, pathological stage such as extracapsular extentsion (ECE) or seminal vesicle invasion (SVI) and positive surgical margins (PSM). However, predictive accuracy of BCR was limited in these studies. Accordingly, substantial efforts have been made to identify novel prognostic markers such as genetic markers to predict BCR after surgical treatment [3, 7–10].

To date, more than 100 single nucleotide polymorphisms (SNPs) have been associated with PCa through genome wide association studies (GWAS) [11]. Many new variants had been published, however not many studies showed PCa related risk variants from Asian populations [11–13] And there are few studies investigate PCa related outcomes using GRS such as BCR except. One study for PCa-associated BCR using combination of genetic information was published [14]. One GWAS in China showed several significant SNPs were associated with PCa susceptibility [15]. Although this report illustrated methods for prediction of cancer risk, PCa related clinical parameters were not included. Other Asian study showed that some PCa variants were significantly associated BCR rates after RP [16]. However, this study was limitation about small analyzed SNP numbers and relative small cohorts.

While SNPs can be easily assessed in blood samples, their clinical use to PCa is still challenging due to large-scale data from genetic analysis. Therefore, the genetic risk score (GRS) was developed, derived and calculated from multiple PCa risk-associated SNPs that have been shown to improve accuracy of disease prediction [17]. Recently, genetic risk assessment studies have evaluated effectiveness of cumulative GRS for PCa risk [18–20], However, no study has used GRS in a model to predict BCR using a GWAS.

In the present study, we performed GRS analysis to predict BCR via a combined clinical-genetic model from a prospective cohort of 912 Korean PCa patients by using Exome chip.

## RESULTS

Among total patients, 212 patients (23.2%) experienced BCR during follow-up duration (median 51-month) (Table 1). The BCR patients had higher initial PSA levels, rates of ECE, rates of SVI, rates of PSM and pathological Gleason scores.

**Table 1:** Baseline characterisitics according to biochemical recurrence after radial prostatectomy

Variables (Mean ± SD)	Total (912)	BCR		*p*-value
		No BCR (700)	BCR (212)	
Mean Age	66.24 ± 6.63	66.33 ± 6.72	65.99 ± 6.37	0.505
Median PSA (ng/ml)	8.30 ± 18.92	7.40 ± 11.32	15.43 ± 31.27	< 0.001
Mean prostate volume (ml)	36.91 ± 16.04	36.44 ± 16.14	38.46 ± 20.45	0.185
Extracapsular extension (%)	303 (33.2)	151 (21.6)	152 (71.7)	< 0.001
Seminal vesicle invasion (%)	94 (10.3)	25 (3.6)	69 (32.5)	< 0.001
Bladder neck invasion (%)	40 (4.4)	11 (1.6)	29 (13.7)	< 0.001
Positive surgical margin (%)	280 (30.7)	151 (21.6)	129 (60.8)	< 0.001
Pathologic stage (%)				< 0.001
pT2	598 (65.6)	542 (77.4)	56 (26.4)	
pT3	301 (33.0)	154 (22.0)	147 (69.3)	
pT4	13 (1.4)	4 (0.6)	9 (4.3)	
Pathology Gleason score (%)				< 0.001
6	62 (6.8)	60 (8.6)	2 (0.9)	
7	712 (78.1)	586 (83.7)	126 (59.4)	
8	42 (4.6)	23 (3.3)	19 (9.0)	
9	96 (10.5)	31 (4.4)	65 (30.7)	
10	0	0	0	

Abbreviations: BCR: biochemical recurrence; SD: standard deviation.

**Figure 1:**
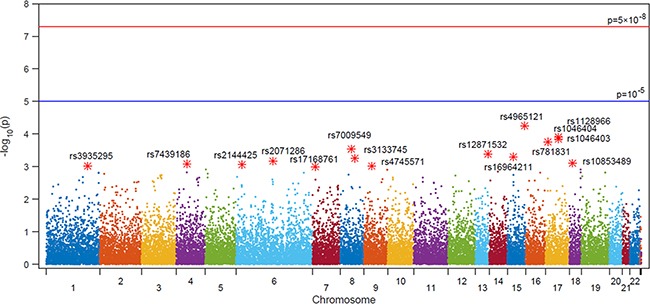
Manhattan plot of SNP association with biochemical recurrence among prostate cancer patients who underwent RP from an analysis of 242,186 single nucleotide polymorphisms using a custom HumanExome BeadChip v1.0 (Illumina Inc.)

The frequency of genotype results according to presence of BCRs were shown in Figure 1. Among the results from 242,186 SNPs, we selected target SNPs with a *p*-value level of 10^–3^. Sixteen SNPs (rs4965121, rs1128966, rs1046404, rs1046403, rs781831, rs7009549, rs12871532, rs16964211, rs3133745, rs2071286, rs10853489, rs7439186, rs2144425, rs3935295, rs4745571 and rs17168761) were significantly associated with BCRs in men after RPs (Table 2). The ORs and significance levels are shown in Table 2.

**Table 2:** Logistic regression analysis of exome array with biochemical recurrence after radical prostatectomy

SNPID	Chr	Alleles	Gene	Minor Allele Frequency		OR (95% CI)	*p*-value
				No BCR	BCR		
rs4965121	15	G > C	.	0.05824	0.1152	2.106 (1.455–3.047)	5.75E-05
rs1128966	17	C > G	NT5C3B	0.1871	0.1083	0.5278 (0.379–0.735)	0.000127
rs1046404	17	C > G	NT5C3B	0.1871	0.1083	0.5278 (0.379–0.735)	0.000127
rs1046403	17	A > G	NT5C3B	0.1871	0.1088	0.5306 (0.381–0.7388)	0.000145
rs781831	17	T > C	ZZEF1	0.3565	0.2593	0.6317 (0.4963–0.804)	0.000176
rs7009549	8	A > G	.	0.5121	0.4124	0.6689 (0.5379–0.8316)	0.000283
rs12871532	13	C > T	.	0.3663	0.2742	0.6536 (0.5156–0.8285)	0.000417
rs16964211	15	G > A	CYP19A1	0.2952	0.2097	0.6335 (0.4895–0.82)	0.000489
rs3133745	8	C > T	C8orf37-AS1	0.2401	0.1613	0.6088 (0.4585–0.8083)	0.000551
rs2071286	6	G > A	NOTCH4	0.1693	0.1019	0.5565 (0.3955–0.7831)	0.000672
rs10853489	18	A > G	.	0.4041	0.4954	1.448 (1.166–1.797)	7.73E-04
rs7439186	4	C > T	AMBN	0.1349	0.2005	1.607 (1.214–2.127)	0.000842
rs2144425	6	A > G	OR12D3, OR5V1	0.3113	0.2281	0.6539 (0.5087–0.8406)	0.000866
rs3935295	1	G > A	PTPN7	0.1607	0.09677	0.5594 (0.3948–0.7927)	0.000959
rs4745571	9	T > C	PRUNE2	0.1712	0.2419	1.545 (1.192–2.004)	0.000966
rs17168761	7	T > C	AGMO	0.3623	0.2765	0.6726 (0.5307–0.8523)	0.000987

Abbreviations: SNP: single nucleotide polymorphism; OR: odds ratio; CI: confidence interval; BCR: biochemical recurrence.

**Figure 2:**
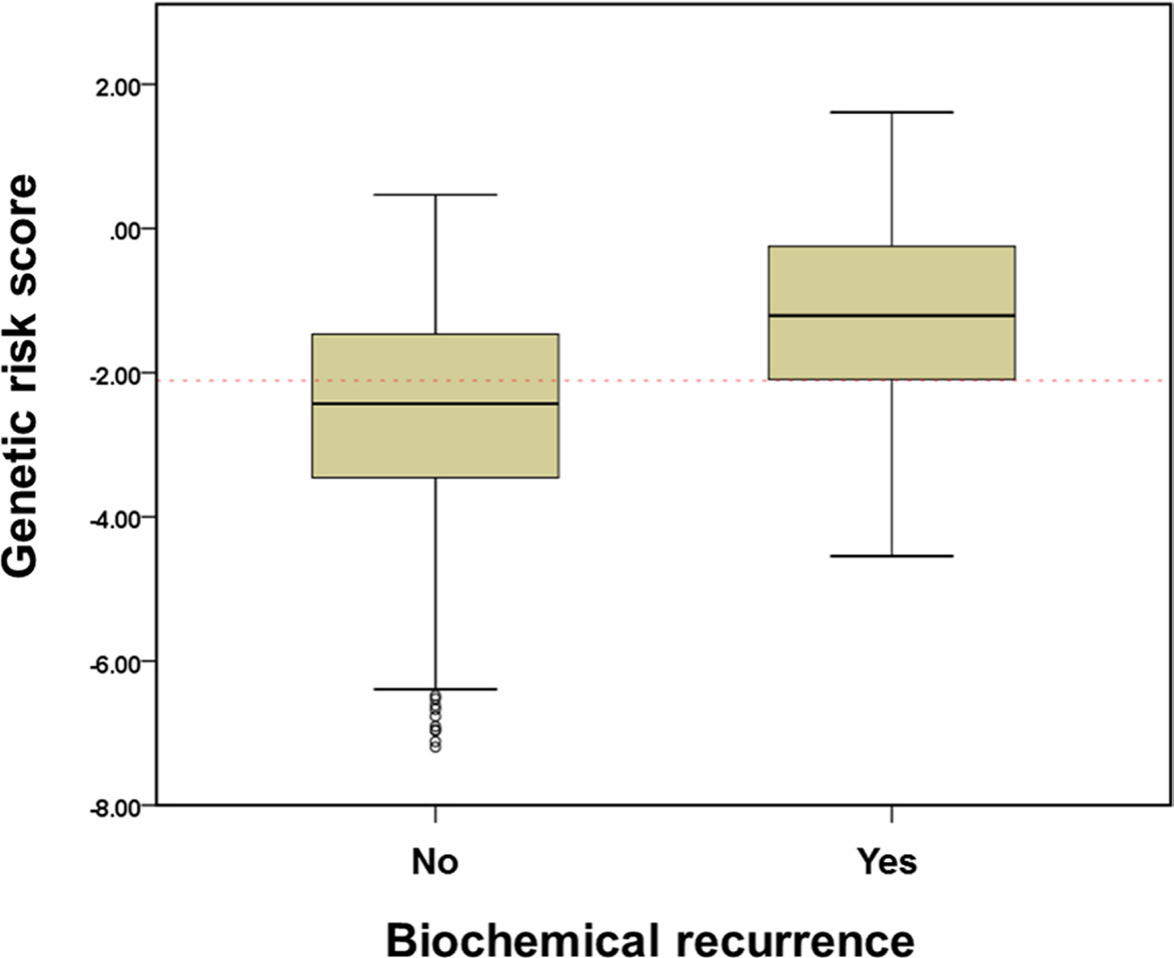
The association of genetic risk score (GRS) with biochemical recurrence after radical prostatectomy among prostate cancer patients GRS was calculated by weighted risk allele count, where risk alleles are weighted by their odds ratios (ORs). GRS = sum of number of risk alleles (0,1,2) × ln (OR).

The median GRS value was –2.10 ± 1.55 in the entire cohort (mean –2.26, range –7.19–1.61). The median GRS calculated in men experiencing BCR was –1.21 ± 1.33 (mean –1.28, range –4.54–1.61). This value was significantly higher than the median score in non-BCR patients (–2.43 ± 1.49, mean –2.56, range –7.19–0.47, *p <* 0.001) (Figure 2).

**Figure 3:**
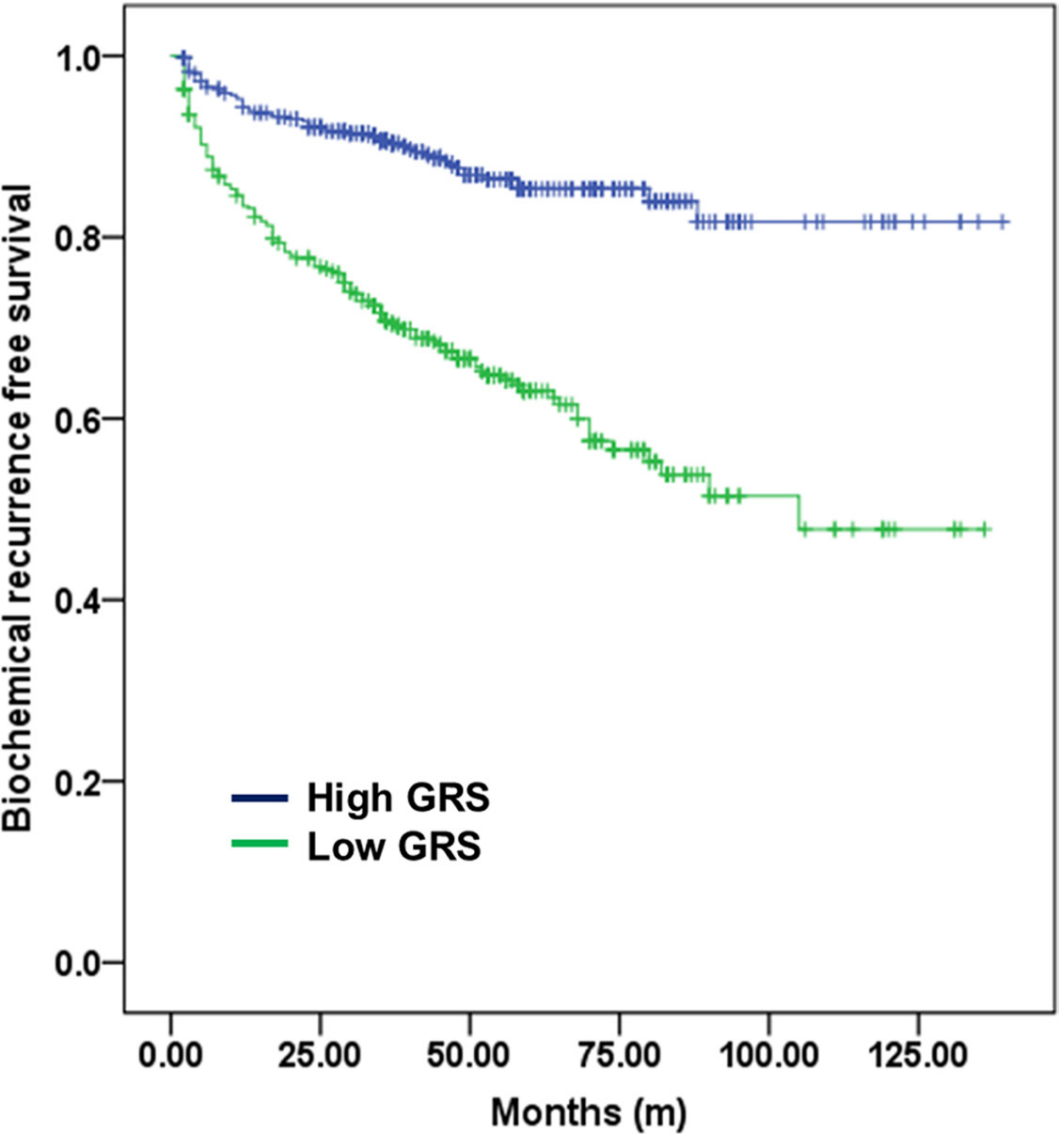
Biochemical recurrence-free survival according to GRS (-2.0) among all patients The high GRS group had a significantly lower BCR-free survival rate than the low GRS group. The 10-year BCR-free survival rate was 76.5% vs. 91.2% among high- and low-GRS patients, respectively (log-rank test *p* < 0.001).

**Figure 4:**
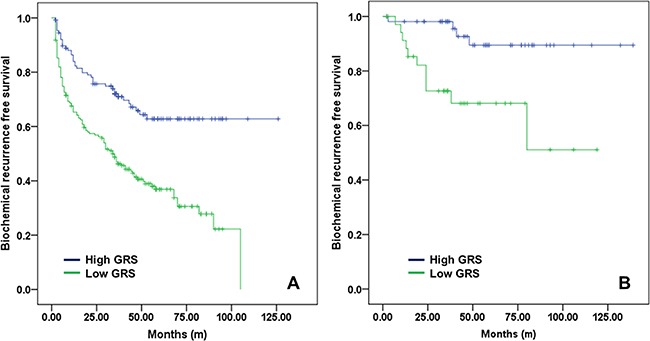
Biochemical recurrence-free survival according to GRS (- 2.0) (**A**) among pathologic T3 patients and (**B**) among pathologic T2 margin-positive patients.

Survival analysis according to GRS value (-2.0) was shown in Figure 3. The high GRS group had significantly lower BCR-free survival rate than the low GRS group. The 10-year BCR-free survival rate was 46.3% vs. 81.8%, respectively (log rank test *p <* 0.001). In subgroup analyses (pathologic T3 and pathologic T2R1 patients), high GRS was also significant factor to BCR outcomes. Among T3 patients (*n* = 314), high GRS patients had 5 year BCR free survival rate was 33.8%, significantly lower in low GRS patients (62.8%) (Figure 4A). Among T2R1 patients (*n* = 90), 5-year BCR-free survival rate was estimated by 87.5% in high GRS vs. 67.9% in low GRS (*p* = 0.001) (Figure 4B).

**Figure 5:**
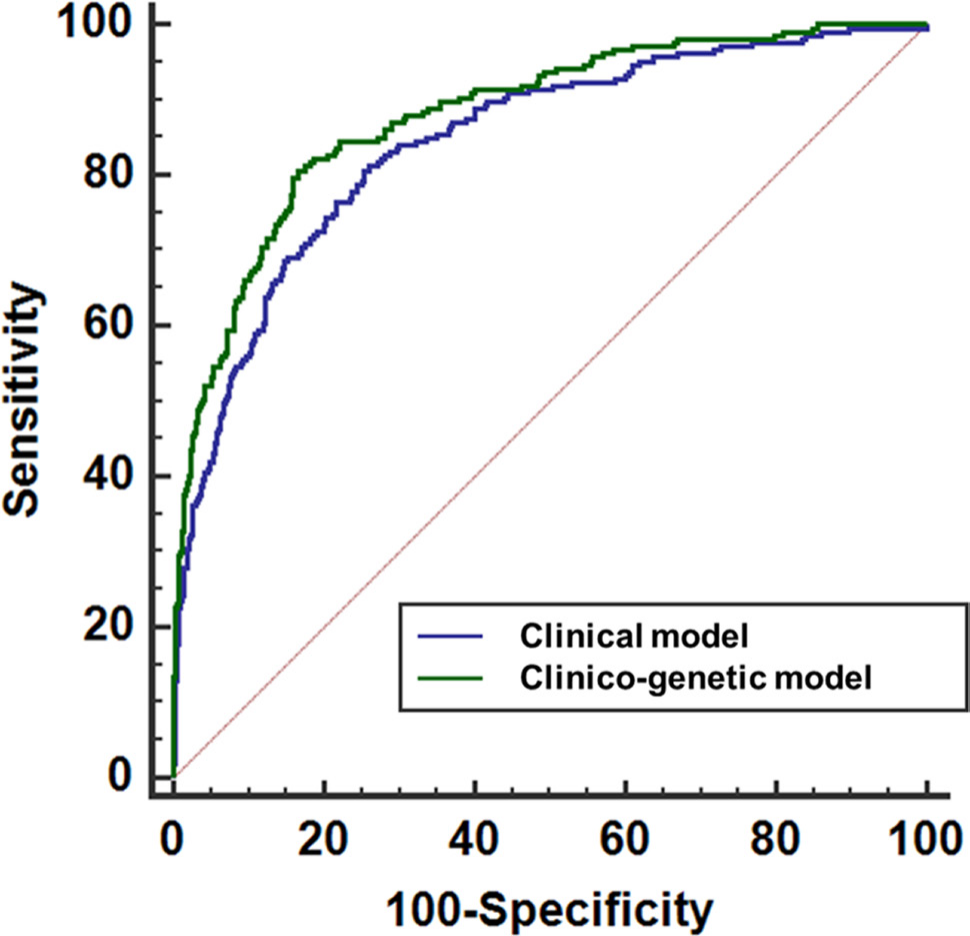
Receiver operating characteristic (ROC) curves of the multivariate logistic regression model devised for biochemical recurrence after radical prostatectomies The blue line corresponds to a clinical model that excludes the genetic risk score (GRS). The green line corresponds to a combined clinico-genetic model that includes GRS.

Separate multivariate Cox proportional hazard models were constructed, both including and excluding GRS. Both models included and adjusted variables such as age, initial serum PSA, pathologic Gleason scores, ECE, SVI and PSM after RP, as shown in Table 3. The GRS was found to be a significant factor after other variables were adjusted (HR: 1.630, 95% CI: 1.454–1.826, *p <* 0.001). The accuracy of prediction to BCR was measured 84.4% in clinical model without GRS, it increased to 88.8% in clinic-genetic model with GRS (95% CI, 0.857–0.900; *p* = 0.0026) (Figure 5).

**Table 3:** Multivariate Cox proportional models of potential predictors for biochemical recurrence of prostate cancer among men who underwent radical prostatectomy and accuracy analysis of established models according to presence of SNP informations

	Clinical model			Clinico-genetic model		
Variables	HR	95% CI	*P* value	HR	95% CI	*P* value
Age (yrs)	0.990	0.968–1.012	0.375	0.990	0.968–1.013	0.400
Initial PSA (ng/ml)	1.003	0.999–1.008	0.117	0.999	0.995–1.003	0.649
Pathologic Gleason score	2.428	1.742–3.385	< 0.001	2.600	1.878–3.600	< 0.001
Extracapsular extensioin	3.090	2.125–4.493	< 0.001	2.847	1.956–4.144	< 0.001
Seminal vesicle invasion	2.637	1.869–3.720	< 0.001	2.461	1.745–3.471	< 0.001
Positive surgical margin	1.972	1.438–2.706	< 0.001	2.010	1.463–2.762	< 0.001
Genetic risk score				1.630	1.454–1.826	< 0.001
Areas under curve of each multivariate models	**0.844**			**0.880**		

Abbreviations: OR: odds ratio; CI: confidence interval; PSA: prostate specific antigen; SNP: single nucleotide polymorphism.

## DISCUSSION

In this study, we found GRS could be additional predictive biomarker to BCR after RP. Using Exome SNP chips containing a large amount of data, we selected 16 SNPs that significantly affected the BCR after radial surgery in PCa. The GRS was developed by the sum of each locus multiplied by the weighted risk allele count. Therefore, GRS was a significant factor after adjusting clinical factors. Although patients achieved similar postoperative and pathological outcomes, GRS from blood samples could accurately predict BCR risk after RP among PCa patients.

In general, PCa patients after RP experienced 5 year BCR free survival rate 80%, 10 year BCR free survival rate 68% [6]. Although high disease free rate, about 35% of patients had experienced BCR. Among them, about 1/3 patients had radiological recurrence and metastatic disease in 8 years after BCR [21]. Therefore, after definitive therapy as RP, it is important to detect the patients who will need adjuvant therapy early. According to the American Society for Therapeutic Radiology and Oncology (ASTRO) and the American Urological Association (AUA) guidelines, physicians should offer adjuvant radiotherapy to patients with adverse pathologic findings at the time of RP (i.e., SVI, PSM, ECE) and should offer salvage radiotherapy to those patients [22]. However, adjuvant therapy after RP could be associated with adverse events such as urinary incontinence. Therefore, a more accurate biomarker or prediction model should be used for early detection or prediction of BCR after RP [23].

Recent GWAS results showed numerous SNPs related with PCa susceptibility. Additionally, a few genetic biomarkers have been associated to PCa aggressiveness [24, 25]. However, the large amount of information from GWAS could not easily be used in clinical situations. Therefore, some simplified calculated methods were introduced. Several customized products such as Oncotype Dx^®^ (Genomic Health Inc, Redwood City, CA, USA), Prolaris^®^ (Myriad Genetics, Salt Lake City, UT, USA) and Decipher^®^ (GenomeDX Biosciences, Vancouver, BC, Canada) using the polygenic risk score concept are currently available for clinical use [26, 27]. One study showed that the cell-cycle progression (CCP) score using prostatectomy specimens had significant prognostic accuracy after controlling for all available clinical and pathologic data among 413 prostate cancer patients. The hazard ratio (HR) for each unit increase in CCP score (range, –1.62 to 2.16) was 2.1 (95% CI, 1.6 to 2.9). With adjustment for Cancer of the Prostate Risk Assessment post-surgical (CAPRA)-score, the HR was 1.7 (95% CI, 1.3 to 2.4). In our study, we constructed a GRS by GWAS analysis using an Exome chip. We found that the HR per unit of GRS was 1.786 (95% CI, 1.604–1.989, *p <* 0.001) in univariate analysis and 1.630 (95% CI, 1.454–1.826, *p <* 0.001) in multivariate methods for prediction of BCR. And another advantage is that our calculated GRS was obtained easily from blood serum.

Several previous studies that combined SNPs and clinical factors to predict PCa outcomes have been published. One study showed 3 SNPs (rs1447295, rs10993994 and rs7920517) were related to BCR in PCa [16]. They adjusted the clinicopathological factors (age, serum PSA, PSM and stage), however they used only 20 SNPs in small 320 patients. Another study also showed precise model for prediction of BCR after RP among 703 patients [28]. They selected 83 SNPs to genotype analysis, and 3 SNPs could be potential marker to predict BCR.

Although these studies showed interesting results to establish more accurate model by adding genetic factors, they also had limitation to relative small patient numbers and genotype field. To overcome these limitations, we prospectively collected many patients and used Exome chip which contained 242,186 SNPs. The action of SNPs to phenotype could be combination of multiple variants, therefore we used GRS to our analysis.

The strengths of our study were as follows: 1) this study was a GWAS-based large-scale study using the HumanExome BeadChip (242,186 SNPs); 2) this study prospectively enrolled patients who underwent RP in a single institution and therefore had similar pathologic analysis by a single pathologist; 3) the GRS was applied to clinical outcome of PCa; 4) a combined clinical-genetic model was created to predict BCR, thereby providing significantly higher accuracy level compared with a model using clinical features alone; and 5) this study was the first conducted in the Korean population. However, the present study had several limitations as well, including its relative small sample size from the perspective of a GWAS. Although all patients included in this study underwent RP in a single institution, 912 patients was a relatively small number. Another limitation was the lack of cancer-specific and all-cause mortality data. Although this study had an intermediate-term follow-up period (median 51.2 months), the amount of cancer-specific and all-cause mortality was relatively small. Therefore, we excluded these factors from the current analysis. Another limitation was there was no validation cohort to confirm of genomic analysis due to our study had unique cohorts who had genomic data, underwent RP and followed-up for BCR in single institution, we could not find relevant validation cohorts. And we also had limitation which there was no multiple testing validated data. The predictive power of GRS has increased by adding as many variant in model, we used this method. The previous many studies used GRS for clinical application, they had same limitation due to several study barriers [18, 29, 30]. After validation, we can build genetic biomarker to predict survival outcomes by simple blood sampling. Combined with genetic markers and clinical factors, we predict PCa outcome more precisely. Furthermore, we will focus the functional mechanism from genotype to phenotype in near future by molecular work.

In conclusion, we detected 16 SNPs that were significant predictors of BCR by analysis using Exome SNP chips. With that information, we developed a GRS-based tool to predict BCR after RP for prostate cancer. The GRS was an important and significant factor for the prediction of BCR. After combination of clinical factors, the combined clinico-genetic model using GRS had a greater predictive power for BCR.

## MATERIALS AND METHODS

### Ethics statement

After approval of institutional review board (SNUBH Institutional Review Board; B-1312/232-302), our study was performed. And we followed the rule of Declaration of Helsinki. The written informed consents were obtained from all participants.

### Study population

The 1,002 male PCa patients were prospectively included in this study from November 2003 to July 2015. All patients’ blood samples were collected prospectively to genotype analysis. We excluded the patients who underwent neo-adjuvant hormonal therapy and/or radiation therapy before RP. The patients whose follow up duration less than 1 year also excluded in this study. After then, total 912 PCa patients were selected in this analysis. All patients had complete clinical records for PCa including BCR data.

### Pathological evaluation and definition of BCR

All specimens of biopsy and RP were processed according to the Stanford protocol [31]. Single uro-pathologist analyzed all specimen. A definition of BCR was two consecutive rises in PSA > 0.2 ng/ml after RP [32].

### Exome genotype and quality control (QC)

The patients’ samples were processed using the Exome chip (HumanExome BeadChip 12v1-1, Illumina, Inc.; San Diego, CA). The 99.706 % markers were successfully genotyped (242,186/242,901). The average call rate was calculated 99.987%. Sample quality control was carried out to exclude samples with genotyping rates < 95%, heterozygosity, and cryptic relatedness. Markers were excluded based on the following criteria: 1) monomorphic in our samples, 2) missing call rate > 5%, 3) significant deviation from the Hardy-Weinberg equilibrium (*P <* 1.0 × 10^–6^). The 912 subjects remained after quality control was taken forward for subsequent analysis.

### SNP analysis methods

Genotype results were analyzed to calculate OR to BCR using logistic regression methods. We used the previous established methods [33, 34] to measure relationship between genotype and haplotype distributions. The genotype analysis was done by SAS version 9.1 (SAS Inc.; Cary, NC, USA). The multiple testing was also done by common methods [35].

### GRS formation

GRS was calculated by weighted-risk allele count, where risk alleles are weighted by their respective ORs [36–38]. The target 16 SNPs were selected to make GRS using cut off value of *p <* 0.001. The natural log (ln) of each OR for each SNP from logistic analysis was multiplied by the number of risk alleles (2, 1, or 0). GRS was calculated by the sum of the value for each locus.

### Statistical analyses

All 912 patients were divided two groups according to experience of BCR. The clinic-pathological factors were compared with Mann–Whitney test, chi-squared test and Fisher's exact test. The BCR-free survival rate according to GRS (median value) was compared using the log-rank test. Another Kaplan-Meier analysis was performed in subgroup of pathologic T3 patients and pathologic T2R1 patients among controversial patients to adjuvant therapy. We performed the multi-variate Cox proportional hazard analyses to confirm significance to BCR after RP by controlling age, initial PSA, p-stage, Gleason score and PSM. Another multi-variate model adding the GRS also performed, and compared two separate multivariate model (with or without GRS) by the methods of rea under the curve (AUC) derived by receiver operating characteristic (Mantel-Haenszel method). The statistical analysis was done by SPSS software package version 21.0 (Statistical Package for Social Sciences™; Chicago, IL, USA) and Medicalc software version 11 (Mariakerke, Belgium). A two-tailed *p <* 0.05 was considered to be significant for all analyses.
